# Reduced striatal vesicular monoamine transporter 2 in REM sleep behavior disorder: imaging prodromal parkinsonism

**DOI:** 10.1038/s41598-020-74495-x

**Published:** 2020-10-23

**Authors:** Leah C. Beauchamp, Victor L. Villemagne, David I. Finkelstein, Vincent Doré, Ashley I. Bush, Kevin J. Barnham, Christopher C. Rowe

**Affiliations:** 1 The Florey Institute of Neuroscience and Mental Health, The University of Melbourne, Melbourne, Australia; 2 The Department of Pharmacology and Therapeutics, The University of Melbourne, Melbourne, Australia; 3 Department of Molecular Imaging and Therapy, Austin Health, Melbourne, Australia; 4 Department of Medicine, The University of Melbourne, Melbourne, Australia; 5 The Australian E-Health Research Centre, CSIRO Health and Biosecurity, Melbourne, Australia; 6 Melbourne Dementia Research Centre, The University of Melbourne, Parkville, Australia

**Keywords:** Neuroscience, Parkinson's disease

## Abstract

Motor deficits in parkinsonism are caused by degeneration of dopaminergic nigral neurons. The success of disease-modifying therapies relies on early detection of the underlying pathological process, leading to early interventions in the disease phenotype. Healthy (n = 16), REM sleep behavior disorder (RBD) (n = 14), dementia with Lewy bodies (n = 10), and Parkinson’s disease (PD) (n = 20) participants underwent ^18^F-AV133 vesicular monoamine transporter type-2 (VMAT2) PET to determine the integrity of the nigrostriatal pathway. Clinical, neurophysiological and neuropsychological testing was conducted to assess parkinsonian symptoms. There was reduced VMAT2 levels in RBD participants in the caudate and putamen, indicating nigrostriatal degeneration. RBD patients also presented with hyposmia and anxiety, non-motor symptoms associated with parkinsonism. ^18^F-AV133 VMAT2 PET allows identification of underlying nigrostriatal degeneration in RBD patients. These findings align with observations of concurrent non-motor symptoms in PD and RBD participants of the Parkinson’s Progression Markers Initiative. Together, these findings suggest that RBD subjects have prodromal parkinsonism supporting the concept of conducting neuroprotective therapeutic trials in RBD-enriched cohorts. Ongoing longitudinal follow-up of these subjects will allow us to determine the time-window of clinical progression.

## Introduction

Parkinson’s disease (PD) is associated with a period of latency in which dopaminergic neurodegeneration is occurring, yet no overt signs of parkinsonism are evident. Preservation of nigro-striatal neurons is paramount to delaying the progression of motor impairment. To date, there has been a failure of translation of neuroprotective drugs from preclinical research and there are no approved therapeutics that halt or modify the progression of the disease. Much of this failure can be attributed to an inability to identify at-risk patients in a preclinical/prodromal phase, where neuroprotective agents would be most efficacious. Currently, diagnosis of PD is based on clinical assessment and relies heavily upon the presentation of movement dysfunction. At this stage, it is estimated that there is already an irreversible loss of 50–70% of dopaminergic terminals in the putamen, based on post-mortem studies^1^ and in vivo neuroimaging of patients in the early stages of unilateral motor impairment^2^. This substantial neurodegeneration occurs *before* clinical diagnosis, significantly narrowing the optimal window for treatment. Hence, there is a critical need to develop preclinical/prodromal markers to identify individuals at-risk that can then be enrolled in early neuroprotective clinical trials.

PD is associated with preclinical non-motor symptoms, including REM sleep behavior disorder (RBD), hyposmia, and anxiety^3^. These symptoms are often reported concurrently and together increase the likelihood of identification of preclinical/prodromal PD^4^. RBD is a form of parasomnia in which people ‘act out their dreams’ during the normally paralytic stage of REM sleep. RBD is reported in 40–50% of PD patients^5,6^, and a number of long-term studies have reported that 65–90% of RBD patients will go on to develop a neurodegenerative disorder, predominantly PD, but also dementia with Lewy bodies (DLB) and multiple system atrophy^7–9^, collectively referred to as parkinsonism.

Between the extensive neurodegeneration that occurs before the onset of motor symptoms and the high likelihood of progression from RBD to a parkinsonism, neuroimaging assessment of the presynaptic nigro-striatal pathway in RBD patients may help the early identification of patients that are likely to progress to PD or another form of parkinsonism and provide a clinical marker for enrolment into preclinical trials.

A number of previous studies have demonstrated striatal dopaminergic neurodegeneration in RBD patients using presynaptic dopamine transporter (DAT) neuroimaging. An early study by Eisensehr et al*.* using [^123^I]IPT-single photon emission computerized tomography (SPECT) on 5 patients with chronic RBD and demonstrated a reduced DAT density in the putamen^10^. More recently, Kim et al*.* reported abnormal DAT imaging using [^123^I]FP-CIT in the putamen of three out of 14 RBD patients^11^, and Iranzo et al*.* demonstrated similar DAT findings in the striatum of 14 out of 43 RBD subjects^12^. These previous finding using DAT SPECT demonstrate a loss of integrity of dopamine circuits in the basal ganglia in RBD patients. Compared to DAT SPECT, VMAT2 PET imaging has improved image quality and quantification^13^, as such, we sought to investigate the dopamine system in RBD patients using the high affinity [^18^F]AV-133 PET tracer which selectively binds to VMAT2.

## Results

Patient demographics and clinical data are summarized in Table 1. There was a higher proportion of men in all clinical groups compared with HC, and the RBD patients were younger than HC (63.5 [9.9] vs. 72.9 [5.1] years, *P* = 0.02). As expected, severe cognitive impairment was present in patients with DLB, as well as motor impairment in the DLB and PD groups compared to HC. There were no differences in dementia, cognitive, or motor scores between HC and RBD patients.

**Table 1:** Patient demographic and clinical data.

	Mean (SD)			
	HC	RBD	DLB	PD
Sample size	16	14	9	20
**Demographics**				
Age, years	72.9 (5.1)	63.5 (9.9)*	68.9 (7.9)	67.0 (9.1)
Sex (M/F), no	9/7	13/1	7/2	18/2
Education, years	14.75 (3.4)	12.08 (4.0)	12.67 (2.6)	14.00 (4.3)
Symptom onset	–	7.6 (4.7)	3.03 (1.9)	6.11 (4.4)
Disease duration	–	–	0.87 (1.9)	4.18 (4.0)
**Neurophysiology**				
UPDRS_m_ score_ON_	–	–	11.7 (5.4)*	13.9 (7.4)*
UPDRS_m_ score_OFF_	1.1 (1.1)	1.5 (1.7)	–	18.1 (9.2)*
UPDRS_m_ subscale				
Bradykinesia	0	0	0.9 (0.9)*	2.1 (0.9)*
Rigitidity	0	0	1.0 (0.5)*	1.8 (0.6)*
H–Y score_ON_	–	–	1.93 (0.9)*	1.60 (0.06)*
H–Y score_OFF_	0.1 (0.3)	0.2 (0.4)	–	1.63 (0.6)*
**Neuropsychology**				
MMSE	29.0 (1.0)	29.3 (1.0)	24.4 (2.9)*	29.0 (1.2)
CDR	0	0.1 (0.2)	0.7 (0.4)*	0.2 (0.5)
HADS				
Anxiety	3.4 (2.6)	6.9 (3.3)*	7.8 (4.1)*	5.1 (3.3)
Depression	2.3 (2.9)	4.1 (2.3)	6.0 (3.5)*	5.1 (2.4)*
ACE-R	91.88 (4.9)	92.07 (5.4)	–	–
Trail Making Test				
Trail A	35.00 (12.8)	32.29 (12.6)	–	–
Trail B	79.44 (27.7)	71.23 (29.0)	–	–
Grooved Pegboard				
Dominant	80.6 (12.5)	83.71 (13.7)	–	–
Non-dominant	95.27 (19.7)	98.93 (30.0)	–	–
Sniffin’ Sticks	9.9 (1.5)	8.2 (2.7)*	–	–
Mayo sleep Questionnaire				
Q1 response (Y/N)	0/16	14/0	–	–
General alertness rating	9.33 (1.3)	7.50 (1.3)*	–	–

*Significantly different from HC (*P* < 0.05), bold values indicate significance

Patients with RBD and DLB had a higher anxiety score than HC (6.9 [3.3] and 7.8 [4.1] vs. 3.4 [2.6], *P* = 0.02, respectively). Further motor, cognitive, olfactory, and sleep examinations were conducted in the HC and RBD groups only. There was no difference between HC and RBD patients in cognition (ACE-R and Trail Making Test), or motor function (Grooved Pegboard), but RBD patients demonstrated impaired olfaction on the Sniffin’ Sticks (8.2 [2.7] vs. 9.9 [1.5], *P* = 0.035). Using an informant-based Mayo sleep questionnaire, *none* of the HC informants responded affirmatively to Q1 (n = 16) (Q1: have you ever seen the patient appear to “act out his/her dream” while sleeping?), and *all* of the RBD informants responded affirmatively (n = 14). Furthermore, RBD patients had decreased alertness, reflected by a decrease in the general alertness score (7.50 [1.3] vs. 9.33 [1.3], *P* = 0.0007, for RBD and HC, respectively) (Table 1).

**Figure 1:**
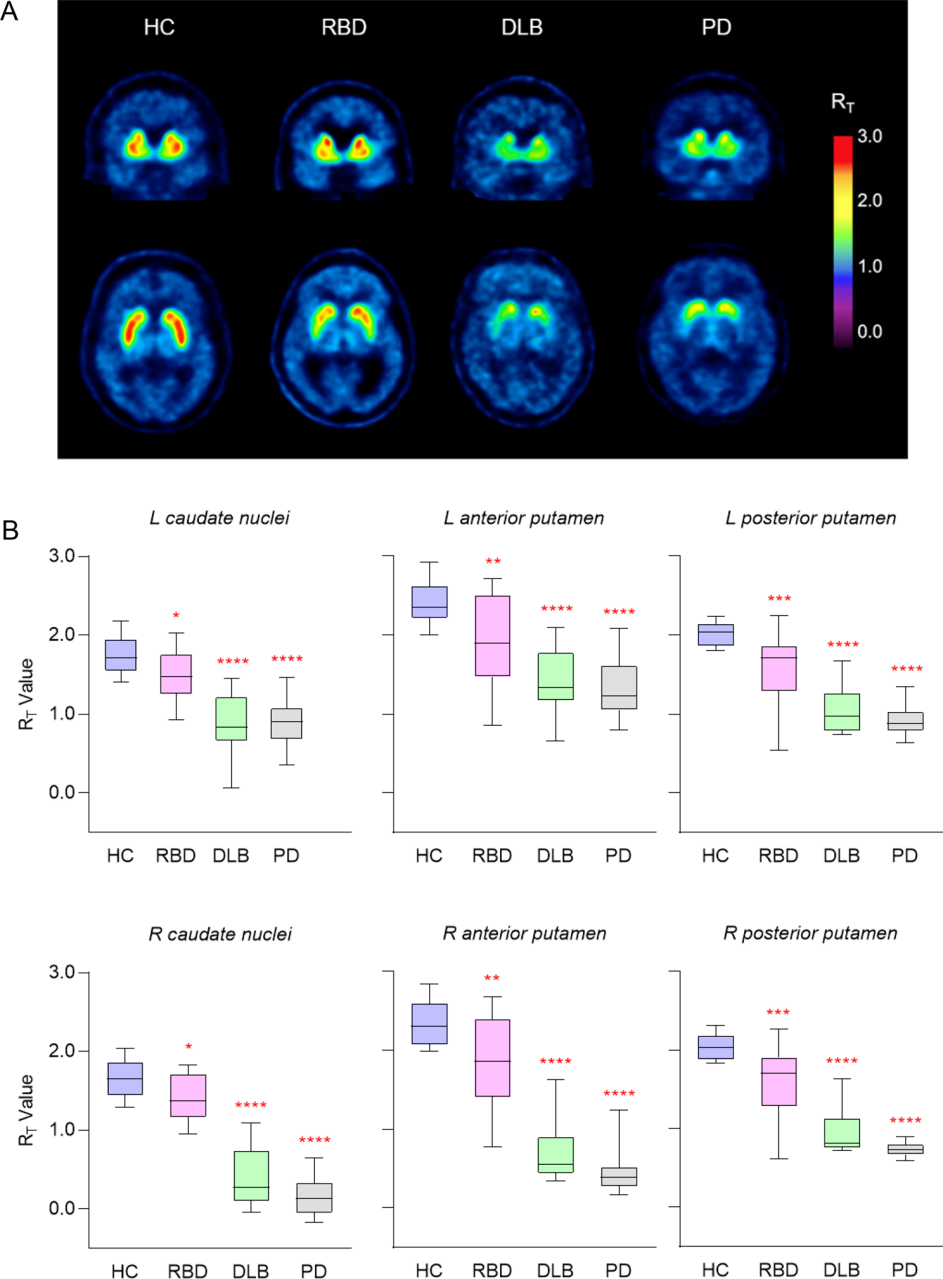
RBD patients have reduced [^18^F]AV-133 binding in the caudate nuclei and putamen. (**A**) Representative [^18^F]AV-133 tissue ratio (R_T_) positron emission tomography images. (**B**) [^18^F]AV-133 positron emission tomography R_T_ values from volumes of interest analysis. Box lines represent median and interquartile range, whiskers represent minimum and maximum values. *Significantly different from HC. *P < 0.05, **P < 0.01, ***P < 0.005, ****P < 0.0001.

Visual inspection showed lower [^18^F]AV-133 binding in RBD, DLB, and PD patients (Fig. 1A). There was a significantly lower R_T_ in all groups compared to HC in the left caudate nuclei, right caudate nuclei, left anterior putamen, right anterior putamen, left posterior putamen, and right posterior putamen (Fig. 1B). There was no significant difference in the anterior midbrain between HC and RBD, however, there was a significant reduction of midbrain R_T_ in the DLB and PD patients. PD patients had significantly lower [^18^F]AV-133 binding compared to RBD patients in all brain regions. (Table 2). There were three RBD participants who had [^18^F]AV-133 binding within the normal range (HC mean ± SD).

**Table 2:** Regional R_T_ values for [^18^F]AV-133 PET.

	Mean (SD)							
	HC	RBD	*P *(vs. HC)	*P *(vs. PD)	DLB	*P *(vs. HC)	PD	*P *(vs. HC)
L caudate nuclei	**1.76** (0.2)	**1.47** (0.3)	0.01	< 0.0001	**0.87** (0.4)	< 0.0001	**0.88** (0.3)	< 0.0001
R caudate nuclei	**1.66** (0.2)	**1.39** (0.3)	0.01	< 0.0001	**0.39** (0.4)	< 0.0001	**0.16** (0.2)	< 0.0001
L anterior putamen	**2.40** (0.3)	**1.90** (0.6)	0.004	0.0011	**1.39** (0.4)	< 0.0001	**1.33** (0.4)	< 0.0001
R anterior putamen	**2.34** (0.3)	**1.82** (0.6)	0.005	< 0.0001	**0.71** (0.4)	< 0.0001	**0.45** (0.3)	< 0.0001
L posterior putamen	**2.02** (0.1)	**1.56** (0.5)	0.0006	< 0.0001	**1.04** (0.3)	< 0.0001	**0.93** (0.2)	< 0.0001
R posterior putamen	**2.04** (0.2)	**1.56** (0.5)	0.0006	< 0.0001	**0.95** (0.3)	< 0.0001	**0.73** (0.1)	< 0.0001
Anterior midbrain	**0.74** (0.1)	**0.61** (0.1)	0.12	< 0.0001	**0.34** (0.3)	< 0.0001	**0.33** (0.2)	< 0.0001

**Figure 2:**
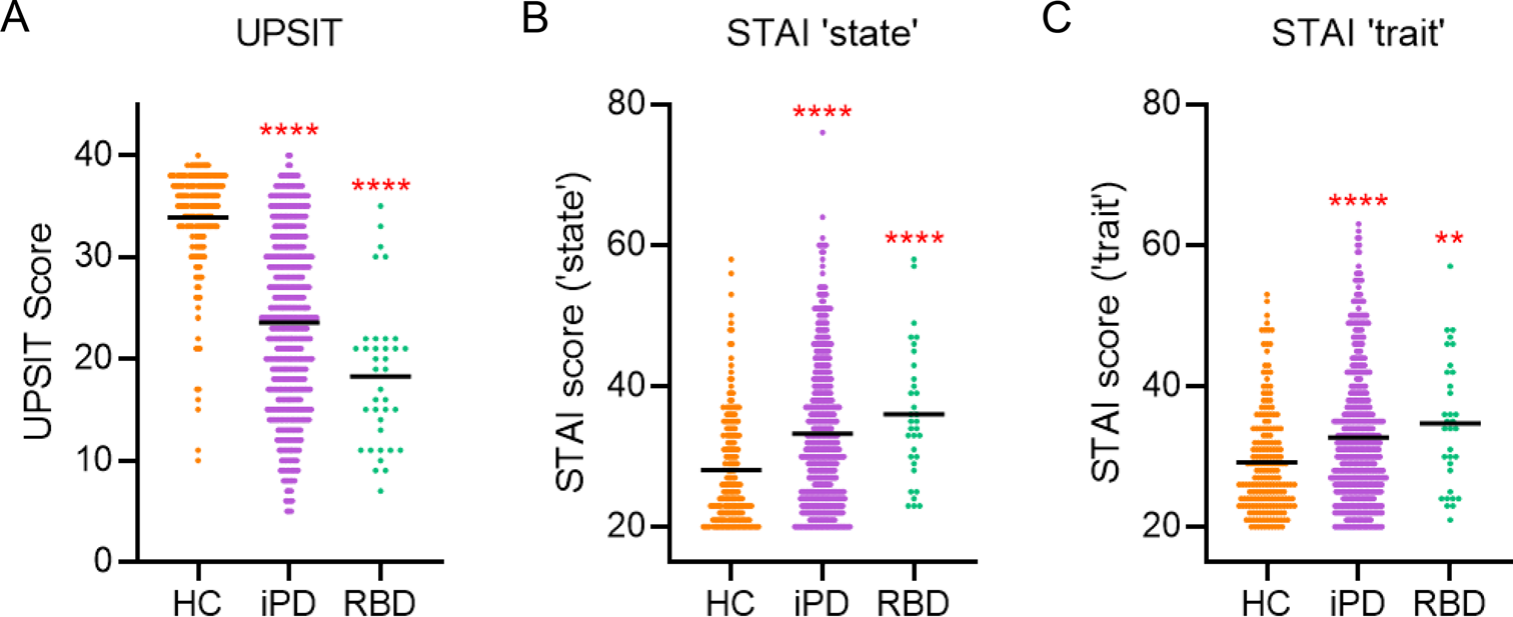
RBD participants from the PPMI have concurrent hyposmia and increased anxiety. (**A**) Olfactory data of HC (n = 197), iPD (n = 492), and RBD (n = 37). (**B**) ‘State’ anxiety of HC (n = 200), iPD (493), and RBD (n = 33). (**C**) ‘trait’ anxiety data of HC (n = 200), iPD (n = 493), and RBD (n = 33). *Significantly different from HC. **P < 0.01, ****P < 0.0001.

The presence of both hyposmia and anxiety in the RBD group from this study is aligned with observations from the large PPMI dataset. The PPMI data, including participants with iPD (n = 492, 55.1% female) and RBD (n = 37, 14.9% female) were compared with HCs (n = 197, 56.0% female). The RBD group was significantly older (70.2 ± 6.4 years) than the iPD and the HC groups (61.7 ± 9.9 years, *P* < 0.0001; 60.8 ± 11.2 years, *P* < 0.0001; respectively). There was hyposmia and increased anxiety in the iPD (UPSIT: 23.57 [8.5], *P* < 0.0001; STAI_state_: 33.20 [10.1], *P* < 0.0001; STAI_trait_: 32.66 [9.5], *P* < 0.0001) and prodromal RBD groups (UPSIT: 18.30 [7.0], *P* < 0.0001; STAI_state_: 36.00 [9.3], *P* < 0.0001; STAI_triat_: 34.67 [9.1], *P* = 0.001) compared to HC (UPSIT: 33.87 [5.1], STAI_state_: 28.09 [7.9]; STAI_trait_: 29.15 [7.5]) (Fig. 2).

## Discussion

This study demonstrates the utility of ^18^F-AV133 VMAT2 PET to indicate the loss of integrity of the nigrostriatal pathway in the striatum of RBD patients in the absence of a classical Parkinson’s motor phenotype. All of the RBD participants held a clinical diagnosis of RBD plus had a positive response to the core Q1 on the Mayo Sleep Questionnaire, which has a sensitivity of 100% and a specificity of 95% in detecting RBD^14^. Furthermore, these patients have anxiety and olfactory deficits, which are recognized symptoms associated with PD^3^. It has been previously shown that there is more severe degeneration in the posterior putamen, followed by the anterior putamen and the caudate nucleus in PD^15^, and this pattern is observed in the RBD patients reported here. Lower VMAT2 levels in the striatum is concordant with findings from a smaller previous study using [^11^C]dihydrotetrabenazine^16^, and these findings recapitulate reduced presynaptic dopamine transporter (DAT) levels using single-photon emission computed tomography in RBD populations^17^. The advantage of assessing VMAT2 over DAT is that while both reflect nigrostriatal degeneration, VMAT2 is less susceptible than DAT to pharmacological challenges and compensatory changes associated with the loss of dopaminergic neurons^18^.

This study demonstrates the concurrence of three prevalent non-motor symptoms with clear reduced VMAT2 levels in disease-associated brain regions, suggesting an increased likelihood that these patients have preclinical/prodromal parkinsonism. To confirm the concurrence of non-motor symptoms was not an artifact of this study, we analysed olfactory and anxiety scores in the large PPMI dataset and confirmed the presence of anxiety and hyposmia in both iPD and prodromal RBD groups.

Attempts at developing disease-modifying therapies have ended in failure, attributable to the substantial loss of axons/synapses at the time of clinical diagnosis. Testing protective treatments where advanced neuronal loss has already occurred dooms these approaches to failure. Ideal subjects are those where nigrostriatal degeneration has begun but not progressed to the point of irreversible damage and profound motor impairments (prodromal PD). RBD patients represent ideal candidates for recruitment into clinical trials for neuroprotective/disease-modifying drugs due to early nigrostriatal neurodegeneration and their increased likelihood of developing clinical PD. Our results suggest that these RBD subjects have prodromal parkinsonism and support the concept that trials of neuroprotective treatments need to be tested in RBD-enriched cohorts.

The main limitations of the present study are the relatively small number of participants and the lack of polysomnography to confirm the extent of RBD. It is also important to note that [^18^F]AV-133 VMAT2 PET may not be reflective of the true extent of nigro-striatal degeneration, as mechanisms such as sprouting of terminals may be compensating for the progressive neuronal/terminal loss^19,20^. ^18^F-AV133 VMAT2 PET allows identification of underlying nigrostriatal degeneration in RBD patients. Ongoing longitudinal follow-up of these subjects will allow to determine the time-window of clinical progression.

## Methods

### Participants

Fourteen patients with clinically diagnosed RBD, along with 10 DLB, 20 PD, and 16 healthy controls (HC) were included in the study. Written informed consent was obtained from all participants, and from a legal guardian of the participants with DLB and PD. The RBD group were referred to the study with a clinical RBD diagnosis, which was the only inclusion criteria for RBD subject recruitment. The participants’ bed partners completed the Informant Mayo Sleep Questionnaire (IMSQ), of which the core question (Q1) on the IMSQ has a sensitivity of 100% and a specificity of 95% in detecting RBD^14^. Data from the DLB and PD patients has been previously published^15^. Approval for the study was obtained from the Austin Health Research Ethics Committee. With regards to dopaminergic medications, 15 patients with PD were receiving carbidopa-levodopa; two selegiline; two, levodopa and benserazide and one, pramipexole. Three DLB patients were receiving carbidopa-levodopa and two, levodopa and benserazide.

### Clinical data

The neurological evaluation of participants included duration of disease and/or symptoms, the Hoehn–Yahr score (H–Y), the motor subscale (Section III) of the Unified Parkinson’s Disease Rating Scale (UPDRS_m_) both on and off medication (medication stopped 24 h prior to examination, and resumed just before the PET scan). The neuropsychological evaluation included the Mini-Mental State Examination (MMSE), Clinical Dementia Rating scale (CDR), and Hospital Anxiety and Depression Scale (HADS) for all groups. HC and RBD participants underwent Trail Making Test, Grooved Pegboard assessment, Addenbrook’s Cognitive Assessment (ACE-R), Sniffin’ Sticks (*MediSense, Netherlands*) and participants bed partners completed the Informant Mayo Sleep Questionnaire.

### Imaging procedures

^18^F-(+)Fluropropyldihydrotetrabenazine (^18^F-AV-133) was synthesized using previously described methods^21^. Each participant underwent a 20-min static acquisition (4 × 5 min frames) at 120–140 min after injection of ~ 250 MBq of [^18^F]AV-133. The sorted sinograms were reconstructed using a 3D RAMLA algorithm. As described earlier^15^, volumes of interest (VOIs) were placed over the standard space MRI over the caudate nucleus, anterior and posterior putamen, midbrain and primary visual cortex by an operator blind to clinical diagnosis. Volumes of interest were then transferred onto the individual [^18^F]AV-133 PET images.

### Image analysis

Tissue ratios (R_T_) of the caudate, anterior and posterior putamen and the anterior midbrain were generated using the primary visual cortex, a region relatively devoid of monoaminergic terminals, as reference region^22^.

### Parkinson’s Progressive Marker Initiative (PPMI) analysis

The PPMI is an observational multicentre study including clinical assessments of olfaction using the University of Pennsylvania Smell Identification Test (UPSIT), and anxiety using the State-Trait Anxiety Inventory (STAI). For the purposes of the data presented in this paper, only a subset of the entire PPMI cohort were analysed including HC, diagnosed iPD, and participants recruited with prodromal RBD. Participating PPMI sites received approval from an ethical standards committee and written informed consent was obtained from all participants.

### Statistical analysis

Statistical comparison of normal data between HC and the three disease groups was performed using a Dunnet test. Non-normal data were analyzed using the Kruskal–Wallis one-way ANOVA. Statistical comparison between HC and RBD groups only was conducted using an unpaired Student’s T test. PPMI data were analysed using a Kruskal–Wallis one-way ANOVA, significance refers to a difference to the HC group. Data were corrected for age-differences. Statistical analysis was performed using GraphPad Prism version 8.0.0 for Windows and significance for each analysis was defined as *P* < 0.05. Data are presented as mean [SD].

### Ethical approval

All methods were carried out in accordance with relevant guidelines and regulations laid out by the Declaration of Helsinki, under ethics approval from the Austin Health Research Ethics Committee.

## Data Availability

All data generated or analysed during this study are included in this published article.
